# Metabolic regulation of triacylglycerol accumulation in the green algae: identification of potential targets for engineering to improve oil yield

**DOI:** 10.1111/pbi.12523

**Published:** 2016-01-23

**Authors:** Elton C. Goncalves, Ann C. Wilkie, Matias Kirst, Bala Rathinasabapathi

**Affiliations:** ^1^ Plant Molecular and Cellular Biology ProgramHorticultural Sciences DepartmentInstitute of Food and Agricultural Sciences, University of FloridaGainesvilleFLUSA; ^2^ Soil and Water Science DepartmentInstitute of Food and Agricultural Sciences, University of FloridaGainesvilleFLUSA; ^3^ School of ForestryUniversity of FloridaGainesvilleFLUSA

**Keywords:** acyltransferases, algae, biofuel, fatty acids, lipid, lipid droplets, nutrient starvation, transcription factors, triacylglycerol

## Abstract

The great need for more sustainable alternatives to fossil fuels has increased our research interests in algal biofuels. Microalgal cells, characterized by high photosynthetic efficiency and rapid cell division, are an excellent source of neutral lipids as potential fuel stocks. Various stress factors, especially nutrient‐starvation conditions, induce an increased formation of lipid bodies filled with triacylglycerol in these cells. Here we review our knowledge base on glycerolipid synthesis in the green algae with an emphasis on recent studies on carbon flux, redistribution of lipids under nutrient‐limiting conditions and its regulation. We discuss the contributions and limitations of classical and novel approaches used to elucidate the algal triacylglycerol biosynthetic pathway and its regulatory network in green algae. Also discussed are gaps in knowledge and suggestions for much needed research both on the biology of triacylglycerol accumulation and possible avenues to engineer improved algal strains.

## Introduction

As a consequence of world population growth reaching an estimated 11 billion by the end of the century (Gerland *et al*., 2014), global energy demand is expected to increase by 37% by 2040 (EIA, 2014). Such a challenge to modern society is aggravated by the finite nature of ubiquitously used fossil fuels, in which the reserves are reportedly diminishing and crude oil prices are constantly fluctuating (Hu *et al*., 2008; Moody *et al*., 2014). The use of fossil fuels, such as petroleum, coal and natural gas, accounts for 85% of the global energy consumption and has raised major environmental concerns. Increasing amounts of greenhouse gas emissions are thought to significantly contribute to environmental damage, affecting global climate and ocean acidification (Greenwell *et al*., 2010; Solomon *et al*., 2009). In addition, climate change negatively affects agriculture, resulting in diminished yields due to droughts, heavy precipitation and high winds, and concomitant increase in food prices (Greenwell *et al*., 2010). Also, the uneven distribution of fossil fuels in the world, highlighted by the presence of 63% of global petroleum reserves in the Middle East, creates an economical unbalance and geopolitical concern (Demirbas and Demirbas, 2011).

The resulting energy security and environmental concerns regarding the use of fossil fuels have led policymakers and researchers to focus their efforts on developing alternative, renewable energy feedstocks with lower environmental impact (Foley *et al*., 2011; Greenwell *et al*., 2010; Ho *et al*., 2014; Wijffels and Barbosa, 2010). Currently, a mere 13% of the world's energy consumption is renewable, and only 10% is derived from biomass (i.e. biological raw materials) (Ho *et al*., 2014). While the development of multiple renewable energy sources, such as cellulosic ethanol, solar, wind and nuclear, is needed to meet the future world energy demand, extraction of high‐energy compounds from algal biomass is a promising alternative needing scientific research and development.

## The potential of using algae as a biofuel feedstock

Microalgae are autotrophic, single‐cell photosynthetic organisms that can reach high lipid content (Chisti, 2008; Hu *et al*., 2008; Markou and Nerantzis, 2013; Medipally *et al*., 2015; Scott *et al*., 2010) that have long been explored as a biofuel feedstock.

In the past decade, microalgae regained researchers' attention due to some species' ability to rapidly grow on open ponds or closed systems (photobioreactors) on land otherwise unsuitable for farming, thus not competing for arable land like oil‐seed crops, their potential for year‐round cultivation, growth in saline water, recycled municipal wastewater and landfill leachate, coproduction of high‐value compounds along with biofuels, and most importantly, their ability to accumulate impressive amounts of lipids under some conditions (Chen *et al*., 2011; Chisti, 2007; Edmundson and Wilkie, 2013; Farooq *et al*., 2015; Markou and Nerantzis, 2013; Moody *et al*., 2014; Pittman *et al*., 2011; Wei *et al*., 2013). Several studies have shown that lipid contents of 20–50% per cell dry weight (CDW) are commonly observed in microalgae (Chisti, 2007; Demirbas and Demirbas, 2011; Griffiths and Harrison, 2009; Mata *et al*., 2010; Powell and Hill, 2009; Singh *et al*., 2011), reaching up to 80% CDW in some species under certain stress conditions (Chisti, 2007, 2008).

Early studies investigating the potential of different sources of feedstocks for replacing conventional transportation fuels showed that terrestrial oil crops, such as canola, soybean and corn, would require up to 1540M hectares to meet 50% of the current U.S. transportation needs. Only 5.4M hectares dedicated for algal cultivation would be necessary to meet the same need (Chisti, 2007, 2008). Most remarkably, theoretical calculations conducted in the same studies suggested that a highly superior oil productivity per hectare could be achieved with microalgae compared to terrestrial oil crops (Chisti, 2007). Part of this is due to the exceptionally fast biomass doubling time of some microalgae (as short as 3.5 h) and their superior photosynthetic efficiency, converting up to 10% of captured light into biomass, compared to 0.5% in conventional terrestrial plants (Costa and De Morais, 2011; Smith *et al*., 2010).

A reduction of up to 50% in carbon dioxide emissions can be achieved in the burning of biodiesel compared to conventional petrol‐based diesel (Brown and Zeiler, 1993; Costa and De Morais, 2011). In addition, roughly 183 tons of carbon dioxide are fixed during the production of 100 tons of algal biomass (Chisti, 2008). In an ideal scenario, in which biodiesel itself and methane produced from the anaerobic digestion of algal residues are used for powering the needs of the production process, photosynthesis‐derived microalgal biodiesel could be considered carbon neutral (Chisti, 2008; Merchant *et al*., 2012).

Despite the recent progress in microalgal research, technical, economic, storage, supply and political barriers still prevent the development and commercialization of microalgal biodiesel (Demirbas and Demirbas, 2011). Although recent technology evaluations have demonstrated the commercial potential for microalgal biofuels in the longer term (Stephens *et al*., 2010), cost‐competitiveness with petroleum products have not been achieved to date (Reijnders *et al*., 2014). Specifically, most microalgae reach the highest lipid contents under unfavourable environmental or stress conditions, which are not conducive to high growth rate and biomass productivity (Hu *et al*., 2008; Markou and Nerantzis, 2013). As a consequence, the organisms' metabolic responses to different stress conditions need to be investigated, and the knowledge applied towards developing strategies to achieve higher algal biomass and lipid productivity in large‐scale production systems.

## Oil accumulation in green algae

Freshwater and marine algae from different taxa (e.g. Chlorophyceae, Trebouxiophyceae, Bacillariophyceae, Eustigmatophyceae) respond to stress conditions by altering their metabolism and accumulating high amounts of neutral lipids and other compounds, such as carbohydrates and secondary metabolites (Griffiths and Harrison, 2009; Griffiths *et al*., 2012; Markou and Nerantzis, 2013; Merchant *et al*., 2012). To date, the algal oleaginous trait has been extensively studied primarily in the model green algae *Chlamydomonas reinhardtii*. Studies in *Nannochloropsis*,* Chlorella* and diatom species have also been of great contribution for elucidating pathways, putative regulatory networks and genetic engineering approaches (Boyle *et al*., 2012; Guarnieri *et al*., 2011; Hockin *et al*., 2012; Li‐Beisson *et al*., 2015; Liu and Benning, 2013; Nguyen *et al*., 2011; Yu *et al*., 2011). However, the signalling pathways by which nutrient stress triggers triacylglycerol (TAG) accumulation, the magnitude of nutrient deficiency required for induction, and the interactions between growth and lipid content under stress conditions are still poorly understood (Adams *et al*., 2013; Park *et al*., 2015; Schmollinger *et al*., 2014). Here we review the most recent research on networks of genes and proteins responsible for changes to carbon flux and redistribution of lipids under nutrient‐limiting conditions.

## Nutrient starvation affects carbon partitioning

**Figure 1:**
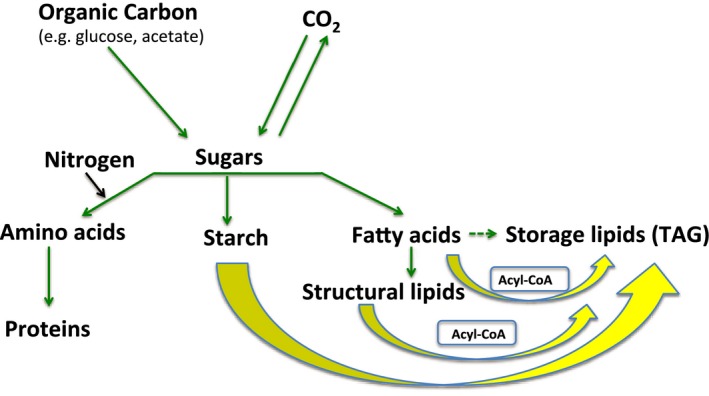
Proposed carbon flux in mixotrophic green algae. Green arrows represent the carbon (C) flux under nitrogen (N)‐replete conditions. Yellow arrows represent redirected C flux under N starvation. Dotted arrow represents a minor contribution to total C flux under N‐replete conditions. The boxes contain the expected intermediate metabolites used for triacylglycerol (TAG) synthesis under N‐starvation conditions.

Gene expression, lipid profiling, ultrastructural, and radiotracer studies have indicated that TAG accumulation in lipid droplets (LDs) under N starvation in green algae is due to (1) increased *de novo* synthesis of TAG from acyl‐CoA, (2) recycling of acyl moieties from the degradation of membrane lipids into TAG and (3) increased carbon flux towards glycerol‐3‐phosphate and acyl‐CoA for fatty acid synthesis (Fan *et al*., 2011, 2012; Goncalves *et al*., 2013; Miller *et al*., 2010). Upon N starvation, a redirection in the flux of atmospheric carbon fixed through photosynthesis and organic carbon sources supplied to the medium, such as glucose, is expected from the end products of primary metabolism (i.e. proteins, structural membrane lipids, starch) to TAG synthesis in most green alga (Figure 1). In fact, the knockdown of a *Chlamydomonas* citrate synthase, which incorporates acetyl‐CoA into the citric acid cycle hence competing with fatty acid synthesis, led to a major increase (169%) in TAG levels (Deng *et al*., 2013), providing strong evidence for the hypothesis of substrate competition for TAG synthesis in green algae. Because the extent to which the carbon flux is affected by nutrient deprivation varies in different species (Hu *et al*., 2008), we infer that there is high genetic diversity for this trait in microalgae.

## Starch turnover upon N starvation

As TAG synthesis is directly affected by carbon partitioning, many studies have investigated the effect of blocking starch biosynthesis as a way to divert carbon flux and increase TAG synthesis. Indeed, multiple studies in both *Chlamydomonas* and *Chlorella* species have shown that blocking starch biosynthesis results in increased lipid accumulation (Li *et al*., 2010; Ramazanov and Ramazanov, 2006; Wang *et al*., 2009; Work *et al*., 2010). While a 30‐fold increase in LD content after 48 h of N starvation in the presence of acetate has been reported in the *Chlamydomonas* wall‐less mutant *sta6* (impaired in ADP‐glucose pyrophosphorylase), the induction in its wild‐type counterpart was only 15‐fold (Wang *et al*., 2009). A subsequent transcriptome study in *sta6* and its parental and complemented strains suggested an increased carbon flux towards hexose‐phosphate in *sta6*, presumably due to an unexpected up‐regulation of the glyoxylate and gluconeogenesis pathways (Blaby *et al*., 2013). In contrast, others have found that the inhibition of the glyoxylate cycle and gluconeogenesis during N starvation leads to increased availability of acetyl‐CoA for fatty acid synthesis (Miller *et al*., 2010; Park *et al*., 2015). Blaby *et al*. (2013) argue that *sta6* acetate consumption might be a consequence of increased activity in acetyl‐CoA synthetase and its product redirection towards TAG synthesis, as starch synthesis is blocked. In a recent time‐course, system‐level study in mixotrophic *Chlamydomonas*, the measurement of transcripts, proteins and metabolites indicated that starch is initially accumulated in the early stage (30 min) of N starvation and degradation starts during the formation of LDs (4–6 h of N starvation) (Park *et al*., 2015). Similarly, an ultrastructural study in mixotrophic *Chlorella* UTEX29 revealed the presence of both starch granules and LDs in the early stage of N starvation (3 h), but only LDs were observed upon prolonged N starvation (48 h) (Goncalves *et al*., 2013). In contrast, a study on multiple *Chlamydomonas* starch biosynthesis mutant strains (including *sta6*) revealed no TAG over‐accumulation upon N starvation compared to its wild‐type direct progenitors (Siaut *et al*., 2011). Although previous studies suggested starch carbon flux redirection to TAG as a promising strategy for increasing lipid productivity in green algae, substantial differences found in commonly used *Chlamydomonas* laboratory strains and the confounding conclusions resulting there from demonstrate the complexity of the control in carbon re‐allocation in N‐starved microalga (Blaby *et al*., 2013; Siaut *et al*., 2011).

## The effect of supplying organic carbon to microalgae

The supplementation of microalgae with organic carbon sources has been used not only as a way to increase lipid productivity in biofuel‐relevant *Chlorella* species (Heredia‐Arroyo *et al*., 2010; O'Grady and Morgan, 2011; Wan *et al*., 2011), but also as an attempt to study the effects of N starvation in different algal species. A supply of unlimited carbon to the cells decreases the secondary responses triggered by changes in photosynthetic rate (Schmollinger *et al*., 2014).

In mixotrophic (i.e. with CO_2_ and organic carbon sources) *Chlorella*, a significant increase in lipid productivity was observed upon supply of glucose, acetate or glycerol, when compared to autotrophic and heterotrophic growth conditions (Heredia‐Arroyo *et al*., 2010). In addition, another study in glucose‐fed *Chlorella* revealed the induction of a heteromeric acetyl‐CoA carboxylase (ACCase), the enzyme catalysing the first committed step in fatty acid synthesis, and repression of the large subunit of carbon‐fixing enzyme ribulose‐1,5‐bisphosphate carboxylase/oxygenase (RUBISCO) during lipid accumulation, suggesting a reduced role of photosynthesis in mixotrophic conditions (Wan *et al*., 2011). These results suggest that mixotrophy is a promising strategy for large‐scale algal cultivation. Although the price of raw materials such as glucose would make such a strategy cost‐prohibitive and compete with food production, there is potential for the use of glycerol and acetate, by‐products of biodiesel and biohydrogen production, respectively (Heredia‐Arroyo *et al*., 2010).

In *Chlamydomonas*, independent studies revealed that adding acetate to the medium led to a boosting effect in lipid accumulation under N starvation conditions and, consequently, an ‘obese’ phenotype (Fan *et al*., 2012; Goodenough *et al*., 2014; Goodson *et al*., 2011). Moreover, Fan *et al*. (2012) showed that lipid accumulation under N starvation conditions in a wild‐type strain is highly dependent upon acetate availability, as increased oil contents were observed when higher concentrations of acetate were available. In agreement with these observations, gene expression analysis of N‐starved *Chlamydomonas* revealed the down‐regulation of glyoxylate and gluconeogenesis pathways, indicating that the conversion of acetate to glucose was redirected into a more direct incorporation of acetate into fatty acids (Miller *et al*., 2010).

## Carbon–nitrogen interconnection

Nitrogen as an essential macronutrient is required for the synthesis of many algal biomolecules, such as amino acids, nucleic acids, and photosynthetic pigments (Hockin *et al*., 2012). The requirement of N‐containing enzymes for C fixation suggests a close metabolic interconnection between the two macronutrients (Schmollinger *et al*., 2014). Several studies have shown that N depletion leads to a drastic reduction in the synthesis of photosynthetic proteins followed by reduction in carbon fixation, and eventually, a chlorotic phenotype (Msanne *et al*., 2012; Plumley and Schmidt, 1989; Valledor *et al*., 2014). The reduction in photosynthetic rate and antennas has been considered as a mechanism to avoid oxidative cellular damages (Valledor *et al*., 2014). One of the proposed underlying principles of this C/N nutrient relationship is that the excess carbon fixed from photosynthesis is channelled into storage molecules, such as TAG, when insufficient amounts of N are available for protein synthesis and cell growth (Scott *et al*., 2010). Transcriptome analysis of the marine green algae *Nannochloropsis* also points to a progressive redirection in the flux of carbon precursors from protein and carbohydrate synthesis to glycerolipid synthesis during N starvation conditions (Li *et al*., 2014). In fact, the supply of excess carbon, either as CO_2_ or as organic forms, induces rapid cell growth with concomitant elevated N consumption, resulting in increased C/N ratio (Reijnders, 2013). After *Chlorella sorokiniana* cultures supplied with high doses of exogenous CO_2_ were exposed to N starvation, enzymes in the Calvin cycle, glycolysis (which converts the intermediate glycerol 3‐phosphate from Calvin cycle into pyruvate) and putative transporters that direct pyruvate to the plastid (e.g. Bile acid:Na^+^ symporter) were induced compared to N‐replete cultures. This suggested that an enhanced CO_2_ assimilation rate could increase carbon flux towards fatty acid synthesis (Sun *et al*., 2015). Therefore, manipulating the culture C/N ratio by increasing the C source without altering N might be a promising strategy for mimicking the processes of N starvation and boosting lipid production in microalgae.

Additional studies in *Chlamydomonas* under N starvation conditions indicated that chlorosis and protein degradation are results of N recycling mechanisms to cope with the decreased cellular C/N ratio, presumably protecting the cells from oxidative stress due to overreduction of the photosynthetic electron transport (Li *et al*., 2012b; Schmollinger *et al*., 2014).

## Elucidating the algal pathway to glycerolipid synthesis

A great improvement in the elucidation of the glycerolipid biosynthetic pathway in green algae has been achieved in recent years due to the increasing interest for its use as a biofuel feedstock (Khozin‐Goldberg and Cohen, 2011). Although the evolutionary diversity of green algae suggests that lipid metabolism in different taxa should be diverse (Liu and Benning, 2013), key lipid synthesis enzymes have been identified by comparative studies with well‐characterized fungal and higher plant models (Li‐Beisson *et al*., 2015; Merchant *et al*., 2012; Riekhof, 2009; Riekhof *et al*., 2005). Moreover, the changes in transcriptome/proteome/metabolome profiles and ultrastructural, radiotracer and physiological studies in N‐starved *Chlamydomonas, Chlorella* and *Nannochloropsis* species have been vital for the elucidation of the TAG biosynthesis and degradation pathways in green algae (Blaby *et al*., 2013; Boyle *et al*., 2012; Goncalves *et al*., 2013; Guarnieri *et al*., 2011; Li *et al*., 2012b; Miller *et al*., 2010; Park *et al*., 2015; Schmollinger *et al*., 2014; Yoon *et al*., 2012).

## *De novo* synthesis of TAG in green algae

The first major step in the *de novo* synthesis of TAG in green algae starts in the chloroplast, where the conversion of acetyl‐CoA into malonyl‐CoA is catalysed by acetyl‐CoA carboxylase (ACCase), the rate‐limiting enzyme in fatty acid biosynthesis (Reverdatto *et al*., 1999). Next, the conversion of malonyl‐CoA into malonyl‐ACP is catalysed by malonyl‐CoA transacylase. Then, fatty acid synthase (FAS) catalyses the elongation of the acyl chains using malonyl‐ACP and acetyl‐CoA as substrates. The termination of the fatty acid chain elongation is catalysed by different fatty‐ACP thioesterases (FAT), which hydrolyse acyl‐ACP into free fatty acids (FFA). Fan *et al*. (2011) demonstrated that TAG synthesis in N‐starved *Chlamydomonas* is dependent on the *de novo* synthesis of fatty acids in the chloroplast. In the study, cerulenin, a specific inhibitor of the type‐2 fatty acid synthase, strongly inhibited N starvation‐induced TAG accumulation.

The fatty acids (in the form of acyl‐ACP) can be directly used for the sequential acylation of glycerol‐3‐phosphate in the chloroplast, which leads to the production of diacylglycerol (DAG). In the chloroplast, DAG mainly serves as a precursor for photosynthetic membrane lipids, such as galactoglycerolipids, which contribute to more than 50% of the total glycerolipids in normal growth conditions (Li‐Beisson *et al*., 2015). DAG can also serve as a precursor for chloroplastic TAG synthesis in *Chlamydomonas* (Fan *et al*., 2011; Goodson *et al*., 2011). In agreement to that, ultrastructural observations of plastoglobuli structures in the chloroplasts of two *Chlorella* species suggest that the chloroplastic TAG synthesis pathway might be conserved between Chlorophyceae and Trebouxiophyceae clades (Goncalves *et al*., 2013; Orus and Martinez, 1991). Alternatively, the FFAs can be exported into the cytosol, where a long‐chain acyl‐CoA synthetase (LACS) joins the FFA with a coenzyme A, producing acyl‐CoA (Fan *et al*., 2011; Mashek *et al*., 2007). Subsequently, acyl‐CoA can undergo elongation and desaturation steps in the endoplasmic reticulum (ER) (Huerlimann and Heimann, 2013). Both soluble plastid‐localized and ER membrane‐bound fatty acid desaturases (FADs) are present in *Chlamydomonas*, oxidizing single bonds of carbon atoms into double bonds at specific locations of the acyl chain (Li‐Beisson *et al*., 2015). Similar to higher plants, most *Chlamydomonas* fatty acids are polyunsaturated, 16 or 18 carbons‐long (Li‐Beisson *et al*., 2015).

**Figure 2:**
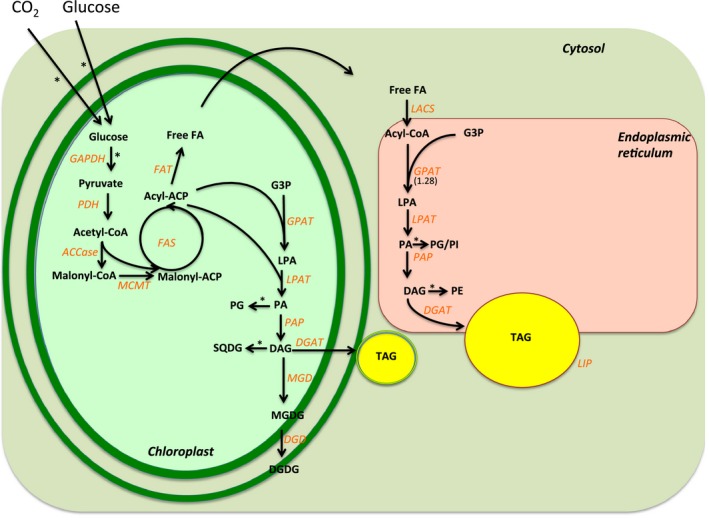
*De novo* triacylglycerol (TAG) biosynthesis in green algae. The enzymes are represented in orange. AADA: alpha amylase domain‐containing protein; ACCase: acetyl‐CoA carboxylase; ACP: acyl carrier protein; CoA: coenzyme A; DAG, diacylglycerol; DGAT: diacylglycerol acyltransferase; DGD: digalactosyldiacylglycerol synthase; FAT: fatty acyl‐ACP thioesterase; FAS: fatty acid synthase; free FA: free fatty acid; G3P: glycerol 3‐phosphate; GPAT: glycerol 3‐phosphate acyltransferase; LACS: long‐chain acyl‐CoA synthetase; LPA: lysophosphatidic acid; LPAT: lysophosphatidic acid acyltransferase; MCMT: malonyl‐CoA:acyl carrier protein malonyltransferase; MGD: monogalactosyldiacylglycerol synthase; PA: phosphatidic acid; PDAT: phospholipid:diacylglycerol acyltransferase; PG: phosphatidylglycerol, SQDG: sulphoquinovosyl diacylglycerol; PI: phosphatidylinositol; PE: phosphatidylethanoalamine; PDH: pyruvate dehydrogenase; PAP: phosphatidic acid phosphatase; TAG: triacylglycerol. *: Additional reactions were omitted for clarity. References for the figure: (Boyle *et al*., 2012; Guarnieri *et al*., 2011; Li *et al*., 2012b; Li‐Beisson *et al*., 2015; Liu and Benning, 2013; Park *et al*., 2015; Yoon *et al*., 2012). (Modified from Goncalves *et al*., 2015).

Several studies have documented that TAG synthesis, through the action of different acyltransferase isoforms, takes place in both the ER‐derived compartments and in the chloroplasts of green microalga (Fan *et al*., 2011; Goodson *et al*., 2011; Li‐Beisson *et al*., 2015). The presence of genes homologous to higher plants' acyltransferases in algae suggests that glycerolipid biosynthesis is a conserved pathway between higher and lower plants (Li‐Beisson *et al*., 2015). Glycerol‐3‐phosphate (G3P) is the carbon ‘backbone’ substrate used for the three sequential acylation reactions that result in TAG formation. First, glycerol‐3‐phosphate acyltransferase (GPAT) catalyses the formation of lysophosphatidate (LPA) by acylation of G3P in the sn‐1 position. Second, lysophosphatidate acyltransferase catalyses the addition of a fatty acid (FA) into the sn‐2 position, giving rise to phosphatidate (PA). Third, phosphatidic acid phosphatase (PAP) catalyses the synthesis of DAG by removing the phosphate group in the sn‐3 position of PA. Finally, diacylglycerol acyltransferase (DGAT) catalyses the acylation of DAG in the sn‐3 position (Huerlimann and Heimann, 2013), resulting in TAG formation. The TAG biosynthetic pathway as currently known in the green algae is shown in Figure 2.

Although algal lipid metabolism gene families are generally smaller than those of higher plants (Riekhof *et al*., 2005), six isoforms of DGAT have been identified in *Chlamydomonas*, in contrast to 2 isoforms in *Arabidopsis* (Ayme *et al*., 2014; Boyle *et al*., 2012; Riekhof *et al*., 2005). Two independent studies have shown that both type‐1 and type‐2 diacylglycerol acyltransferases, DGAT1 and DGTT1, whose subcellular localizations are predicted to the chloroplast and secretory pathway respectively (Li‐Beisson *et al*., 2015), were induced by N starvation (Boyle *et al*., 2012; Miller *et al*., 2010) and also by starvation for sulphur, iron, phosphorus or zinc. All these stress factors trigger TAG accumulation, but to a lesser extent than N starvation (Boyle *et al*., 2012). This suggested that the overexpression of DGAT isoforms could be a way to improve TAG accumulation in algae. Subsequent studies revealed no significant increases in TAG content when three *Chlamydomonas* type‐2 DGAT isoforms were presumably overexpressed in *Chlamydomonas* (La Russa *et al*., 2012). Only DGAT mRNA levels were measured in those lines. Thus, post‐transcriptional and post‐translational mechanisms that could have prevented the successful overexpression of DGAT cannot be ruled out. In fact, overexpression of a type‐2 DGAT in the marine diatom *Phaeodactylum tricornutum* confirmed at the protein level by Western blotting, led to a 35% increase in neutral lipid content without affecting culture growth (Niu *et al*., 2013). Further gene function studies in green algae, including the overexpression of type‐1 DGAT isoforms, might be key to uncover precursor and enzymatic activity bottlenecks. In our efforts to engineer algal strains to increase flux to glycerolipids, a more global genetic engineering approach, such as the overexpression of putative transcription factors controlling multiple steps in TAG synthesis, could be a superior approach.

## Membrane recycling mechanisms and lipid droplet stabilization

Studies conducted in *Arabidopsis* knockout mutants for phospholipid:diacylglycerol acyltransferase (PDAT), which contains a catalytic lipase motif, suggested that increased TAG synthesis is a protection mechanism against free fatty acid‐induced cell death in fast‐growing tissues of plants (Dahlqvist *et al*., 2000; Fan *et al*., 2013). Moreover, the accumulation of TAG in *Arabidopsis* seeds depends on both PDAT and DGAT acyltransferases (AtPDAT and AtDGAT, respectively). While AtDGAT catalyses the direct acylation of DAG, AtPDAT is involved in the acyl‐editing pathway, catalysing the transfer of an acyl group from the sn‐2 position of phosphatidylcholine (PC) to the sn‐3 position of DAG (Zhang *et al*., 2009).

In *Chlamydomonas*, a single PDAT protein (CrPDAT) was detected on purified lipid droplets through a proteomics study (Nguyen *et al*., 2011). The identification and cloning of CrPDAT's gene allowed for further *in vitro* studies that demonstrated the enzyme's broad substrate specificity, including phospholipids, galactolipids, TAG and cholesteryl esters (Yoon *et al*., 2012). The increased expression of CrPDAT's transcript and increase in its corresponding protein levels under N‐starvation conditions, with simultaneous reduction in the concentration of membrane lipids (monogalactosyldiacylglycerol (MGDG), sulphoquinovosyl diacylglycerol (SQDG) and phosphatidylglycerol (PG)), suggested CrPDAT's role in the recycling of membrane lipids upon such stress condition (Boyle *et al*., 2012; Yoon *et al*., 2012). Furthermore, the fact that PDAT‐deficient or null mutants are viable and contain a reduced, but not abolished TAG content points to a functional redundancy between PDAT and DGAT in *Chlamydomonas* (Boyle *et al*., 2012; Yoon *et al*., 2012).

One of the first studies to demonstrate the contribution of recycling of membrane lipids into TAG during N starvation was conducted by Fan *et al*. (2011). Using an elegant approach, in which the *de novo* synthesis of fatty acids was blocked using cerulenin, they tracked the changes in individual lipid classes during a time‐course N‐starvation experiment. A decrease in the membrane lipids such as MGDG and PG, mirrored by an increase in TAG content, suggested that as much as 30% of the total TAG fatty acids have originated from membrane lipids in N‐starved *Chlamydomonas* (Fan *et al*., 2011). Conversely, a reduction of up to 80% in plastidial membrane lipids has been reported in *Chlamydomonas* N‐starved for 48 h (Siaut *et al*., 2011). In another study, the *Chlamydomonas* mutant *pgd1,* defective in a galactoglycerolipid lipase, was isolated in a screening for reduced TAG accumulation under N deprivation (Li *et al*., 2012b). PGD1 was found to be responsible for the hydrolysis of MGDG, significantly contributing to the increased pools of fatty acids that are incorporated into TAG during N deprivation conditions in Chlorophyceae (Li *et al*., 2012b). Similarly, an ultrastructural and radiotracer study in N‐starved *Chlorella* UTEX29 also found membrane recycling to be a major contributor to N‐starvation‐induced TAG accumulation in this member of the Trebouxiophyceae clade (Goncalves *et al*., 2013). These results suggest that this lipid recycling response upon N deficiency is conserved between Chlorophyceae and Trebouxiophyceae species.

Putative lipases and esterases are also likely involved in the recycling of membrane lipids into TAG, contributing to its accumulation in LDs (Merchant *et al*., 2012). It would be expected that, in order for TAG to accumulate, TAG lipases would be repressed under N‐starvation conditions. On the contrary, a transcriptome study found that 27% of the 130 putative lipase‐encoding genes identified were up‐regulated following N deprivation (Miller *et al*., 2010). Surprisingly, another independent transcriptomics study revealed both increased and decreased expression of putative TAG lipases following N deprivation (Boyle *et al*., 2012). As the induction of TAG lipases during TAG accumulation is counterintuitive and wasteful from a metabolism point of view, it is plausible to hypothesize that these lipases catalyse the turnover of membrane lipids rather than TAG (Boyle *et al*., 2012; Miller *et al*., 2010). Moreover, a *Chlamydomonas* gene encoding a lipase, *CrLIP1*, has been identified based on its ability to complement a lipase‐deficient mutant in yeast (Li *et al*., 2012a). Reducing *CrLIP1*'s transcript abundance using artificial miRNA led to a delay in TAG lipolysis after N resupply, which indicated that CrLIP1 indeed facilitates TAG turnover (Li *et al*., 2012a). Likewise, the *Chlamydomonas* mutant *cht7* (*compromised hydrolysis of triacylglycerols 7*), a putative repressor of cellular quiescence, was also impaired in TAG turnover following N resupply (Tsai *et al*., 2014).

After TAG is synthesized, its stabilization into LDs is possible due to a surface layer of polar phospholipids and oleosins, which are the most abundant proteins found in LDs of higher plants (Huang *et al*., 2013). In contrast, studies suggest that a major lipid droplet protein (MLDP), the proposed equivalent of plant oleosins, is the main protein controlling LD biogenesis and size in *Chlamydomonas* (Huang *et al*., 2013; Moellering and Benning, 2010; Nguyen *et al*., 2011). In *Chlamydomonas*, MLDP was the most abundant protein found in the lipid droplet fraction and its partial gene repression using RNA interference led to a significant increase in lipid droplet size without affecting TAG content (Moellering and Benning, 2010)**.**

## Nitrogen‐starvation sensing and transduction

Nitrogen starvation is one of the most commonly used, efficient and reproducible ways to trigger lipid accumulation across different lineages of microalgae, especially in the members of the Chlorophyta clade (Blaby *et al*., 2013; Dong *et al*., 2013; Liu and Benning, 2013; Rodolfi *et al*., 2009; Schmollinger *et al*., 2014). An early study on 55 algal species under photoautotrophic laboratory conditions showed that increased lipid content in species belonging to Chlorophyta correlates well with nitrogen deprivation in the media (Griffiths and Harrison, 2009). In contrast, the same study showed a greater variation in the response to N starvation in species from other taxa. Moreover, the metabolic response to N starvation is remarkably fast, with accumulation of LDs in *Chlorella* being observed as early as 3 h of growth on N‐free medium (Goncalves *et al*., 2013).

In *Arabidopsis*, there is evidence that two nitrate transporters (NRT1.1 and NRT2.1) might act as nitrate sensors in addition to their transport function (Gojon *et al*., 2011). Therefore, despite little experimental evidence, it is plausible that similar transceptors (dual‐function membrane proteins) also play a role in N signalling in microalgae. A study on *Chlorella protothecoides,* initially grown autotrophically and then transferred into a high C/N ratio medium as a way to trigger lipid production, revealed induction in the expression of the ammonium transporter *CpAMT1* during lipid accumulation (Yan *et al*., 2013). While *CpAMT1*'s expression was modulated by ammonium, glutamine and glutamic acid, it was not changed by nitrate availability. The correlation in expression between *CpAMT1* and glutamine synthetase/glutamate synthetase suggested that *CpAMT1* might be one of the N sensors in *C. protothecoides* (Yan *et al*., 2013). This is consistent with the observation that glutamine, the primary metabolite derived from nitrogen assimilation in plants, along with ATP and 2‐oxoglutarate, controls the C/N sensing mechanism and homeostasis in cyanobacteria (Forchhammer, 2004). A phylogenetic study indicated that glutamine signalling, via a plastid‐localized signalling protein, originated in cyanobacteria and is conserved in green algae (Chellamuthu *et al*., 2014).

In recent years, diverse omics approaches (i.e. transcriptomics, proteomics, lipodomics and metabolomics) have been used in an effort to better understand the mechanisms underlying N‐starvation‐induced lipid accumulation in green algae (Blaby *et al*., 2013; Boyle *et al*., 2012; Gargouri *et al*., 2015; Guarnieri *et al*., 2011; Miller *et al*., 2010; Park *et al*., 2015; Schmollinger *et al*., 2014). While these studies have significantly contributed to the elucidation of the algal TAG biosynthetic pathway and some of its underlying principles, very few putative regulators of this metabolic response have been identified. A major breakthrough was the identification of nitrogen response regulator 1 (*NRR1*), a putative transcription factor with a regulatory role in N assimilation and TAG accumulation in N‐depleted *Chlamydomonas* (Boyle *et al*., 2012). *NRR1*'s identification was possible due to its tightly coregulated expression with the acyltransferase *DGTT1*, and the ammonium transporter *AMT1D* following N deprivation. In addition, NRR1 expression was not modulated by other nutrient deficiencies, indicating that its regulatory role is exclusive to N (Boyle *et al*., 2012). Given the fact that mutating *NRR1* led to a 50% reduction in TAG accumulation rather than total inhibition during N deficiency, the presence of additional, yet to be uncovered, key regulators controlling lipid synthesis under N stress is possible. Our recent study has shown a likely novel role for the MYB‐related transcription factor rhythm of Chloroplast 40 (*ROC40*), which was previously implied in the control of circadian rhythmicity in *Chlamydomonas* (Matsuo *et al*., 2008). ROC40 protein was highly induced upon 3 h of N starvation in *Chlorella* UTEX29 and its gene disruption in *Chlamydomonas* led to a significant reduction in total lipids upon 48 h of N starvation (Goncalves *et al*., 2015). Another study identified the triacylglycerol accumulation regulator 1 (*TAR1*), a tyrosine phosphorylation‐regulated kinase implicated in the control of TAG accumulation upon both N and sulphur (S) deficiencies (Kajikawa *et al*., 2015). The mutant *tar1* was isolated in a screen for low TAG accumulation upon S deficiency using TAG fluorescence measurements. While *tar1* accumulated 50% of the TAG content of its wild‐type counterpart upon S deficiency, TAG content dropped to 10% of the level of the wild type upon N deficiency. In contrast to *NRR1*, the mutation in *TAR1* did not affect the expression of key enzymes in glycerolipid biosynthesis (e.g. DGAT, PDAT) and acetate assimilation (e.g. acetate kinases, acetyl‐CoA synthetases), despite the severe phenotype under N‐starvation conditions (Kajikawa *et al*., 2015). Moreover, the commonly observed degradation of chlorophylls and turnover of plastidial membrane lipids (MGDG and DGDG) upon prolonged N deficiency was significantly reduced in the mutant *tar1* compared to the wild type, implying that *TAR1* mediates the degradation of the photosynthetic apparatus upon stress condition. In addition, the arrest in cell division in N‐starved *tar1* suggests its function in regulating the cell cycle. Finally, it is possible that *TAR1's* pleiotropic effect in both N and S deficiency (affecting TAG accumulation, cell division and photosynthesis) might be a result of its phosphorylation activity upon transcription factors controlling downstream reactions. However, the targets of TAR1 remain unknown to date (Kajikawa *et al*., 2015).

More recently, technological advances in combined omics approaches (i.e. systems biology analysis) permitted in‐depth investigations of the early regulatory networks responding to N deficiency in *Chlamydomonas* (Gargouri *et al*., 2015; Park *et al*., 2015). Transcript, protein and metabolite changes revealed a biphasic response in which N deficiency is sensed within 30 min, initially stimulating gluconeogenesis and starch synthesis. Within 4–6 h of N deprivation, the cells transition to a glycolytic stage and lipid biosynthesis occurs concomitantly with reduction in chloroplast membranes and starch (Park *et al*., 2015), in agreement with previous studies (Blaby *et al*., 2013; Goodenough *et al*., 2014; Schmollinger *et al*., 2014). In addition, the rapid metabolic repatterning observed suggests that such changes cannot be explained by modulation in intracellular N or C/N availability. Rather, that appears to be controlled by sensing mechanisms involving transcription factors (TFs) and transcriptional regulators (TRs) (Park *et al*., 2015). A follow‐up analysis by the same group of investigators revealed the transcript and protein modulation of over 400 TFs and TRs following N depletion (Gargouri *et al*., 2015). Major regulatory network hubs controlling lipid synthesis under N‐starvation conditions were elucidated taking into consideration the early and late, up and down‐regulated TFs and TRs.

Despite the insights and resources provided by these studies in the transcriptional regulatory networks in *Chlamydomonas*, few studies have pinpointed the function of specific genes in TAG accumulation in green algae using multiple lines of evidence including biochemical pathway analyses in mutants and engineered strains (Table 1). Such additional studies are needed especially to validate TFs and TRs controlling glycerolipid synthetic pathways in algae.

**Table 1:** Genes with confirmed roles in TAG synthesis and accumulation in lipid droplets in green algae

Gene	Description	Algal species	Functional validation	Effects (compared to parental strains/control conditions)	Role	References
*NRR1*	Nitrogen response regulator 1	*Chlamydomonas reinhardtii*	Insertional gene disruption	52% less TAG content	Putative transcription factor controlling N assimilation and TAG accumulation	Boyle *et al*. (2012)
*DGTT1*	Diacylglycerol acyltransferase 1	*Chlamydomonas reinhardtii*	Heterologous complementation of yeast quadruple mutant carrying deletions in 4 acyltransferases	Rescue of oleic acid sensitivity; restoration of TAG accumulation	Catalyses the acyl‐dependent acylation of DAG, contributing to TAG accumulation upon ‐N	Boyle *et al*. (2012)
*PDAT1*	Phospholipid: diacylglycerol acyltransferase 1	*Chlamydomonas reinhardtii*	Insertional gene disruption Heterologous complementation of yeast quadruple mutant carrying deletions in 4 acyltransferases	25% less TAG Restoration of TAG accumulation Rescue of oleic acid sensitivity	Mediates membrane lipid turnover. Catalyses the acyl‐dependent and independent acylation of DAG into TAG. Uses DAG and membrane lipids as substrates	Boyle *et al*. (2012)
*CIS*	Citrate synthase	*Chlamydomonas reinhardtii*	RNAi gene expression knockdown Gene overexpression	169% increase in TAG level 45% decrease in TAG level	Indirect control of TAG synthesis. Acetyl‐CoA flux is diverted to fatty acid synthesis rather than TCA cycle when CIS activity is disrupted	Deng *et al*. (2013)
*LIP1*	Lipase 1	*Chlamydomonas reinhardtii*	Artificial micro‐RNA gene expression knockdown Heterologous complementation of yeast mutant carrying deletions in 2 major TAG lipases	Delay in TAG lipolysis upon N resupply Restoration of lipase‐deficiency phenotypes	Broad‐specificity lipase that facilitates TAG turnover for rapid cell growth upon N resupply	Li *et al*. (2012a)
*PGD1*	Plastid Galactoglycerolipid degradation 1	*Chlamydomonas reinhardtii*	Insertional gene disruption	Decreased fatty acid flux of plastid lipids to TAG upon ‐N. Reduction by 50% in the ratio of fatty acids incorporated into TAG over total fatty acids in lipid extract upon 3 days of ‐N	Hydrolyses MGDG, contributing to increased pools of fatty acids to TAG synthesis upon ‐N	Li *et al*. (2012b)
*TAR1*	Triacylglycerol accumulation regulator 1	*Chlamydomonas reinhardtii*	Insertional gene disruption	Reduction by 90% in TAG content upon ‐N	Tyrosine phosphorylation‐regulated kinase implicated in the control of TAG accumulation upon both N and S deficiencies	Kajikawa *et al*. (2015)
*PDAT1*	Phospholipid: diacylglycerol acyltransferase 1	*Chlamydomonas reinhardtii*	Artificial micro‐RNA gene expression knockdown Heterologous expression in yeast	Up to 57% reduction in TAG content upon N‐replete conditions and 28% reduction upon 24 h of ‐N Up to a threefold increase in TAG content	Mediates membrane lipid turnover. Catalyses the transacylation of DAG into TAG. Uses DAG and membrane lipids as substrates. Broad‐specificity lipase activity	Yoon *et al*. (2012)
*MLDP*	Major lipid droplet protein	*Chlamydomonas reinhardtii*	RNA interference gene expression knockdown	Average 40% increase in lipid droplet diameter	Most abundant protein in lipid droplets. Controls lipid droplet size. Does not affect TAG content	Moellering and Benning (2010)
*ROC40*	Rhythm of chloroplast 40	*Chlamydomonas reinhardtii*	Insertional gene disruption	Attenuation of N‐starvation induced increase in triacylglycerol level	MYB‐related transcription factor implicated in the regulation of ‐N‐induced TAG accumulation and control of circadian clock	Goncalves *et al*. (2015)^a^

TAG, triacylglycerol; DAG, diacylglycerol; TCA, tricarboxylic acid cycle.

a Studies that used gene disruption, overexpression, and mutant complementation approaches were selected.

## Other stress factors that induce lipid accumulation in algae

While other nutrient deficiencies (e.g. sulphur, iron, zinc, phosphorus) and abiotic stress conditions (different light intensities) have also been reported to trigger lipid accumulation in green algae (Boyle *et al*., 2012; Hu *et al*., 2008), quite contrasting results are observed in the literature, making nitrogen deficiency still the preferred stress condition to study the mechanistic details of TAG accumulation in green algae. For example, phosphorus (P) starvation has led to both increases and decreases (Hu *et al*., 2008; Rodolfi *et al*., 2009) or no effect (Fan *et al*., 2011) in TAG content in different microalgal species. Principal component analysis of P‐ and N‐starvation‐modulated metabolites in *Chlamydomonas* indicates that the deficiency syndrome of each nutrient was quite different. In fact, P‐starvation effects take longer to be observed than N‐starvation effects, presumably due to a quicker mobilization of internal phosphate reserves found in *Chlamydomonas* vacuoles (e.g. pyrophosphate and short‐/long‐chain polyphosphates), temporarily alleviating internal P deficiency (Bolling and Fiehn, 2005). The protein PSR1 (phosphorus‐starvation response), which contains putative myb DNA binding domains, has been implicated in the regulation of phosphorus metabolism in *Chlamydomonas* through mutant analysis, but its connection to lipid accumulation has not been investigated (Wykoff *et al*., 1999). PSR1 transcript levels increased 10‐fold under P‐starvation conditions and its protein levels significantly increased in the nucleus, suggesting it might play a role regulating the expression of P metabolism genes, such as activating low affinity inorganic P transporters under P‐starvation conditions (Wykoff *et al*., 1999).

As discussed earlier, the kinase TAR1 is implicated in the mediation of sulphur (S) and N deficiency responses in *Chlamydomonas*. TAR1 is expected to exclusively regulate lipid metabolism upon S starvation because the only phenotypic difference between the mutant *tar1* and the wild‐type strain was the TAG content, in contrast to previously mentioned multiple phenotypes of *tar1* upon N deficiency. The mechanisms by which TAR1 regulates the responses to S starvation are unknown to date (Kajikawa *et al*., 2015).

The effects of various light intensities in modulating lipid production and fatty acid saturation have also been reported in many algal species (Hu *et al*., 2008; Lichtle, 1980; Napolitano, 1994; Van Wagenen *et al*., 2012). An early ultrastructural study in the freshwater red alga *Cryptomonas rufescens* demonstrated that high light intensity leads to the accumulation of both intracellular starch and lipids and eventual cyst formation, while lipid synthesis alone is induced by N deficiency in this species (Lichtle, 1980). Similar to N starvation, high light intensity typically induces neutral lipid (TAG) accumulation in algae, while low light seems to induce the synthesis of membrane polar lipids. Moreover, low light has been shown to induce the formation of polyunsaturated fatty acids (PUFAs) while high light favours the synthesis of saturated and monounsaturated FAs (Fabregas *et al*., 2004; Hu *et al*., 2008). A recent study in glucose‐fed *Chlorella zofingiensis* showed that, in the presence of light, lipid synthesis is attenuated while starch synthesis and cell proliferation are increased compared to cultures grown in the dark (Chen *et al*., 2015). Based on these observations and on the down‐regulation of several fatty acid biosynthetic genes in cultures grown under light, the authors suggest that, although light stimulates cell proliferation and greater biomass yield, it might attenuate lipid synthesis by triggering a redirection of the carbon flux from lipids to starch (Chen *et al*., 2015).

Finally, the effect of silicon (Si) starvation is as critical for inducing lipid accumulation in diatoms as N starvation is in green algae (Griffiths and Harrison, 2009; Hu *et al*., 2008). Diatoms require large amounts of Si for the biomineralization of their unique cell walls (frustule), and also for cell division and DNA replication (Shrestha and Hildebrand, 2015). An early study has shown that Si starvation not only induces TAG synthesis but also modulates FA profile, with increasing proportions of saturated and monounsaturated fatty acids being observed under Si‐starvation conditions (Roessler, 1988). An average increase from 24 to 41% and as high as 50% CDW in lipid content was observed under Si‐starvation conditions in a study on laboratory‐grown algae (Griffiths and Harrison, 2009). A recent study in wild type and silicic acid transport (SIT) mutant strains of the diatom *Thalassiosira pseudonana* investigated the changes in transcript, protein and lipid abundance upon Si‐replete and Si‐deplete conditions (Shrestha and Hildebrand, 2015). Interestingly, RNA interference and antisense knockdowns in the SIT proteins promoted a faster lipid accumulation under Si‐starvation conditions, suggesting SIT proteins' dual role as transporters and sensors (transceptors) of silicon in diatoms.

## Biological role of TAG accumulation

One of the proposed interpretations for the drastic increase in TAG accumulation in N‐starved green algae is that it is a cellular response for efficiently storing excess energy and assimilated carbon under suboptimal conditions (Greenwell *et al*., 2010; Hu *et al*., 2008; Solovchenko, 2012; Valledor *et al*., 2014; Wan *et al*., 2012). Moreover, this metabolic rearrangement would prevent oxidative cellular damage, therefore maintaining the cell's vitality, as N starvation is known to negatively affect the light harvesting complexes and associated proteins controlling the photosynthetic electron transport (Schmollinger *et al*., 2014). A recent system‐level study in *Chlamydomonas* revealed that N starvation triggers the accumulation of most proteins related to oxidative phosphorylation (Valledor *et al*., 2014), such as NADH dehydrogenase, ATP synthases and cytochrome c oxidase. This response seems to be unique to N starvation, as it is not observed upon other nutrient stress conditions, such as sulphur deprivation (Gonzalez‐Ballester *et al*., 2010), which triggers starch accumulation rather than TAG (Zhang *et al*., 2002). In addition to the proposed ‘efficient energy storage’ hypothesis, others have proposed that TAG serves as a sink for free fatty acids under N‐starvation conditions, preventing lipotoxicity in the cytoplasm (Greenwell *et al*., 2010; Kurat *et al*., 2006; Solovchenko, 2012).

Another plausible biological role that has not been fully investigated to date is that TAG accumulation could confer a *fitness* advantage for the cells under suboptimal nutrient conditions. The altered buoyancy of the cells could facilitate its movement to regions of higher nutrient availability. We propose that studies on algal buoyancy under different nutrient‐depletion modes and the ability of the cells to recover after nutrient resupply would be essential for testing this hypothesis. Furthermore, dietary studies on laboratory‐reared *Daphnia pulex,* a predator of algae*,* showed that when the green algae *Scenedesmus obliquus* was N‐deprived and contained high oil content, *Daphnia* growth rate, size and number of eggs produced were decreased compared to using algae under N‐replete conditions (low oil content) as feed (Groeger *et al*., 1991). Thus, N deprivation in green algae might also confer a *fitness* advantage against predation either by supplying lower N or higher amount of lipids to the predator. Finally, as the number of available algal mutants in lipid biosynthesis rapidly increases (Liu and Benning, 2013), new opportunities are provided to compare the *fitness* of mutants and the corresponding wild‐type strains under different environmental conditions.

## Recovery upon N availability

When the environmental conditions are reversed to normal, N becomes available for the cells and photosynthesis is resumed. During this period, the degradation of TAG provides energy for the fast recovery of vegetative cell growth (Valledor *et al*., 2014). The polyunsaturated fatty acids contained in TAG molecules have been proposed to serve as reservoirs for the rapid synthesis of chloroplast membranes upon N recovery (Khozin‐Goldberg and Cohen, 2011). In algal strains in which starch is also accumulated during N deprivation, the resupply of N in the absence of light induces starch turnover within a few hours to support cell growth, followed by a later induction of TAG turnover (Siaut *et al*., 2011). Several studies have documented the fast disappearance of lipid droplets with concomitant reappearance of plastidial membranes within hours after N resupply (Cagnon *et al*., 2013; Li *et al*., 2012a; Siaut *et al*., 2011).

The effects of N starvation can be easily reversed by adding N to the medium, which has been useful for investigating the algal recovery responses after N resupply in the oleaginous microalgae *Nannochloropsis oceanica* IMET1 (Dong *et al*., 2013). In this study, when nitrate was resupplied after long‐term N starvation (31 days), physiological parameters such as the photochemical energy conversion efficiency of photosystem 2 indicated that the photosynthetic capacity was reversed to normal within 4 days. Moreover, the measurement of protein expression during the recovery phase revealed the induction of proteins related to photosynthesis, glycolysis, tricarboxylic acid cycle and lipid degradation, supporting the physiological observations and suggesting a remobilization of the carbon flux from TAG accumulation to the normal routes of the algal primary metabolism (Dong *et al*., 2013). In *Chlamydomonas*, a mutant named as compromised hydrolysis of triacylglycerols 7 (CHT7) was isolated in a screen for mutants impaired in the recovery after N resupply to N‐deprived cultures (Tsai *et al*., 2014). However, CHT7's role as an N sensor was ruled out as the mutant's regrowth was also compromised following resupply of other nutrients after their deprivation. Rather, transcriptome data suggested that CHT7 is a repressor of cellular quiescence (halt of cellular division) controlling downstream transcriptional programs associated with nutrient deprivation (Tsai *et al*., 2014).

## Regulon engineering for induced accumulation of TAG

Despite major insights provided by recent gene function and systems biology studies, further research will be fundamental to identify the regulatory cascades controlling TAG accumulation under stress conditions in green algae. Recent improvements in genomic data, such as high‐quality gene models, have been made because of the initial efforts put into sequencing the *Chlamydomonas* genome (Blaby *et al*., 2014). In addition, major advancements have been made in the techniques available to interrogate gene function, including chemical and insertional mutagenesis, genome‐editing approaches, RNA interference, transformation markers and fluorescent proteins (Jinkerson and Jonikas, 2015). These approaches, in combination with high‐quality reference genomes, will enable the conduction of much needed in‐depth studies on the mechanistic details of transcriptional and translational control over glycerolipid synthesis and metabolism. This could lead us to new opportunities to engineer superior oil‐accumulating strains. Specific transcription factors, such as NRR1, ROC40, TAR1 and several others recently revealed, also respond to nutrient limitation (Gargouri *et al*., 2015; Park *et al*., 2015). They could be expressed under induced promoters to test their significance in modulating TAG synthesis. While *Chlamydomonas* will be a great resource to solve scientific questions, efforts into technology transfer targeting robust oil‐producing strains that can grow outdoors, in different environments, without being outcompeted by other algae, and resist predators, will be fundamental for the development of the algal biodiesel industry.
